# The retinoblastoma family of proteins and their regulatory functions in the mammalian cell division cycle

**DOI:** 10.1186/1747-1028-7-10

**Published:** 2012-03-14

**Authors:** Shauna A Henley, Frederick A Dick

**Affiliations:** 1 London Regional Cancer Program, Western University, London, Ontario, Canada; 2 Children's Health Research Institute, Western University, London, Ontario, Canada; 3 Department of Biochemistry, Western University, London, Ontario, Canada; 4 Grande Prairie College, Grande Prairie, Alberta, Canada

**Keywords:** Cell cycle, Senescence, Transcription, Cyclin dependent kinase

## Abstract

The retinoblastoma (RB) family of proteins are found in organisms as distantly related as humans, plants, and insects. These proteins play a key role in regulating advancement of the cell division cycle from the G1 to S-phases. This is achieved through negative regulation of two important positive regulators of cell cycle entry, E2F transcription factors and cyclin dependent kinases. In growth arrested cells transcriptional activity by E2Fs is repressed by RB proteins. Stimulation of cell cycle entry by growth factor signaling leads to activation of cyclin dependent kinases. They in turn phosphorylate and inactivate the RB family proteins, leading to E2F activation and additional cyclin dependent kinase activity. This propels the cell cycle irreversibly forward leading to DNA synthesis. This review will focus on the basic biochemistry and cell biology governing the regulation and activity of mammalian RB family proteins in cell cycle control.

## Introduction

The retinoblastoma gene (*RB1*) was first identified based on its mutation in a rare malignancy of the eye [1,2]. Shortly thereafter, viral oncogenes such as human papilloma virus E7, simian virus TAg, and adenovirus E1A, were discovered to target it for inactivation during cellular transformation [3-5]. Based on sequence similarity, and analogous interactions with viral proteins, two other members of the RB family were identified, *RBL1 *and *RBL2 *that code for the p107 and p130 proteins, respectively [6-11]. All three RB family members contain a conserved domain referred to as the 'pocket' that interacts with the LXCXE motif found in viral proteins such as TAg [12]. For this reason the RB family is also frequently called the pocket protein family. Pocket proteins are present and thought to be central to the regulation of proliferation in many diverse organisms [13]. Furthermore, deregulation of cell cycle control in cancer requires the inactivation of their growth regulatory function [14].

In cancer, the *RB1 *gene is most frequently inactivated through alterations to cyclin dependent kinase regulation, however, in specific cancer types such as small cell lung cancer and retinoblastoma it is uniformly abrogated by direct mutation [15,16]. Reports of cancer derived mutations in the other RB family genes are less common, nevertheless, experimental models of cancer using mice that are deficient for these genes indicate that *RBL1 *and *RBL2 *loss can enhance the cancer phenotype in *RB1 *mutant animals [17-20]. This suggests that the pocket protein family has a collective role in cell cycle control and tumor suppression. In most cancers, their ability to regulate the cell cycle is likely bypassed by altering their common upstream cyclin dependent kinase regulators [15]. At the same time, differences in cancer derived mutations between these genes suggest there may be important biological differences within the RB family. Research on the pocket proteins has often followed this paradigm. In some circumstances the RB family of proteins are perceived to function analogously, while in other instances they can have dramatically different functions. In this review the basic biochemical functions of the pocket proteins will be emphasized. To guide readers through the intricacies of this gene family, we will make particular care to emphasize their similarities and contrast their differences.

## The pocket protein family: pRB, p107, and p130

**Figure 1:**
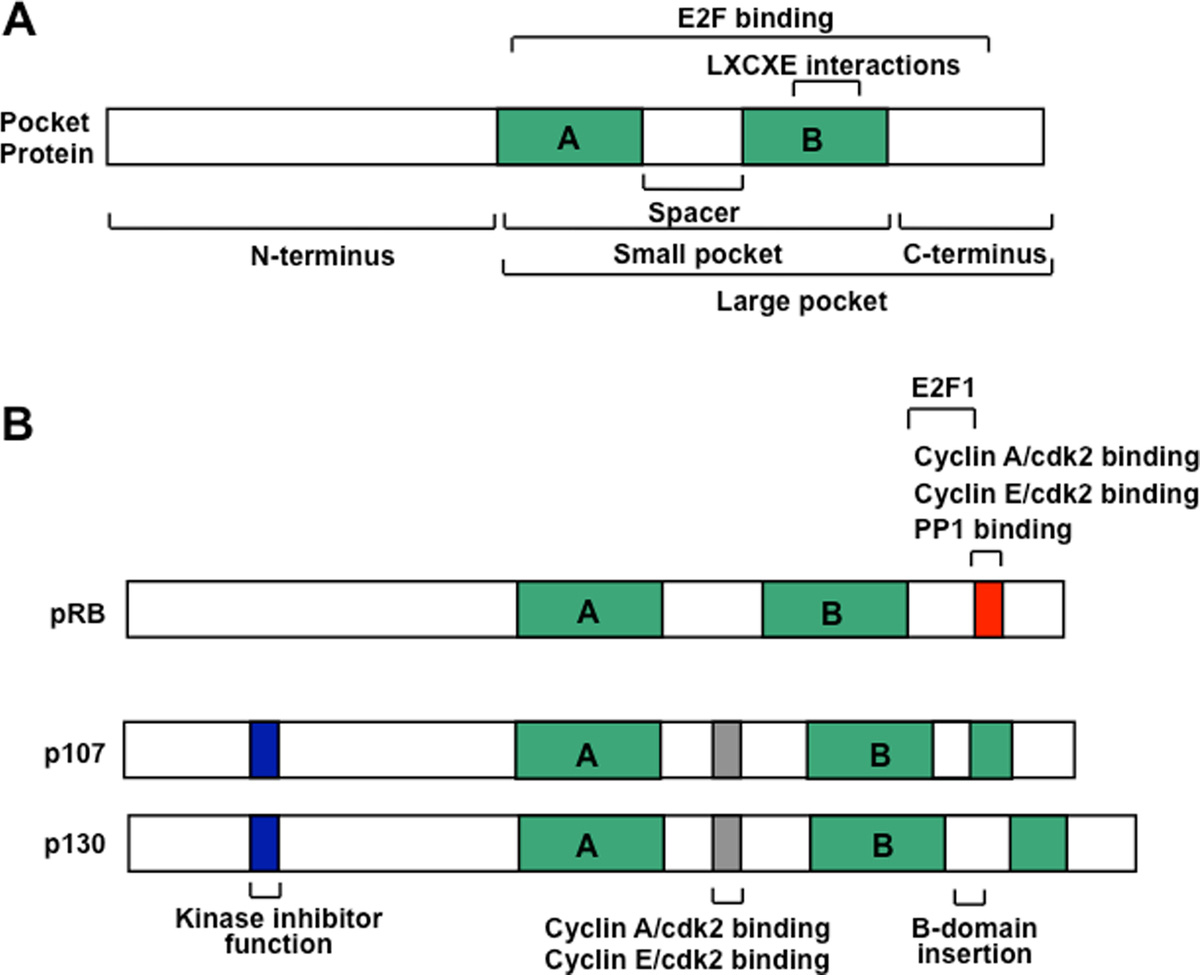
**Schematic representation of pRB, p107 and p130 open reading frames**. (A) The central feature of RB-family proteins is the pocket domain. It was originally defined as the minimal domain necessary to bind to viral oncoproteins such as simian virus TAg through their LXCXE motif, and is denoted as the 'small pocket' in this diagram. The 'large pocket' is the minimal growth suppressing domain of RB-family proteins and it is capable of binding E2F transcription factors as well as viral proteins. (B) Comparison of open reading frame structures of each of the pocket proteins. Note the additional features found in the p107 and p130 proteins, the kinase inhibitory site, the cyclin binding site, and the insertion in the B-domain of the pocket. These provide the most obvious differences between pRB and its relatives p107 and p130.

The RB family members share many structural properties (Figure 1A). The most extensive sequence homology lies in the well-conserved, *small pocket *region, which consists of A and B domains that are separated by a flexible spacer region [21]. These A and B domains each represent a single cyclin fold domain [22] and interact such that the small pocket is self sufficient to form a transcription repressor on its own [23-25]. The small pocket is the minimal fragment of pRB that is capable of interacting with viral oncoproteins, such as E1A and TAg [26]. Even though they are derived from highly unrelated viruses, these viral proteins each contain a peptide motif called LXCXE that is essential for a stable interaction with RB family proteins [4,27-30]. Crystallographic data has revealed that the LXCXE motif contacts a shallow groove on pRB, that is among the most well conserved features among pocket protein family members, and among pocket proteins across species [22]. In addition to the viral proteins, a number of cellular proteins are reported to contain an LXCXE-like motif that allows them to interact with pRB, p107 and p130 [12]. Many of these LXCXE containing proteins possess chromatin regulating activity, or are components of complexes that possess this activity. For these reasons, cellular proteins that contact this region of pocket proteins are generally thought to negatively regulate transcription and this will be described in more detail in the ensuing sections of this review.

The combination of the small pocket and the C-terminal domain has been coined the *large pocket *(Figure 1A), and it is the minimal growth suppressing domain found in RB family proteins [31]. The large pocket fragment is sufficient to interact with E2F family transcription factors and suppress their transcription [32,33]. Interaction with E2Fs is a common feature of RB family proteins that plays a key role in their ability to control proliferation. While E2F interactions are distinct from LXCXE contacts, binding of viral oncoproteins through the LXCXE motif tethers them in close proximity so that a separate region on the viral protein can disrupt binding between pocket proteins and E2Fs [34-36]. This further emphasizes the importance of RB family-E2F interactions because disrupting them is essential for E1A viral oncogene driven transformation [37].

While the overall structure of the pocket domain is well conserved between the three proteins, p107 and p130 are more closely related to each other by sequence similarity than either is to pRB [21]. Surprisingly, despite pRB's prominent description as the central tumor suppressor within this family, and divergence in sequence similarity [17], there are few obvious structural features that it possesses that are missing from p107 and p130 (Figure 1B). Two unique features of pRB that have emerged recently are a docking site used only by the E2F1 transcription factor, and a short peptide region in the C-terminus that is competitively occupied by cyclin/cyclin dependent kinases (CDKs) or protein phosphatase 1 (PP1) (Figure 1B). The region of pRB that mediates these interactions has little obvious sequence divergence from p107 and p130 and the functional basis for these distinct aspects of pRB function will be discussed extensively in later sections. So while there is little to distinguish pRB from its siblings, somewhat surprisingly there are a number of well known features in p107 and p130 that aren't present in pRB. Both p107 and p130 proteins contain insertions in the B domain of their small pockets. In the case of p130, this insert is subject to regulatory phosphorylation to maintain protein stability [38]. Furthermore, p107 and p130 contain longer spacer regions than pRB, and their spacers allow them to interact stably with cyclin dependent kinase complexes [39-41]. Lastly, p107 and p130 contain an N-terminal region that serves to inhibit cyclin dependent kinases [41].

The ability of pocket proteins to use these structural features allows them to interact with a myriad of binding partners to control cell cycle advancement, and perhaps much more. The uses of these interaction sites will be expanded on and discussed in greater detail below.

## The role of pocket proteins in an advancing cell cycle

**Figure 2:**
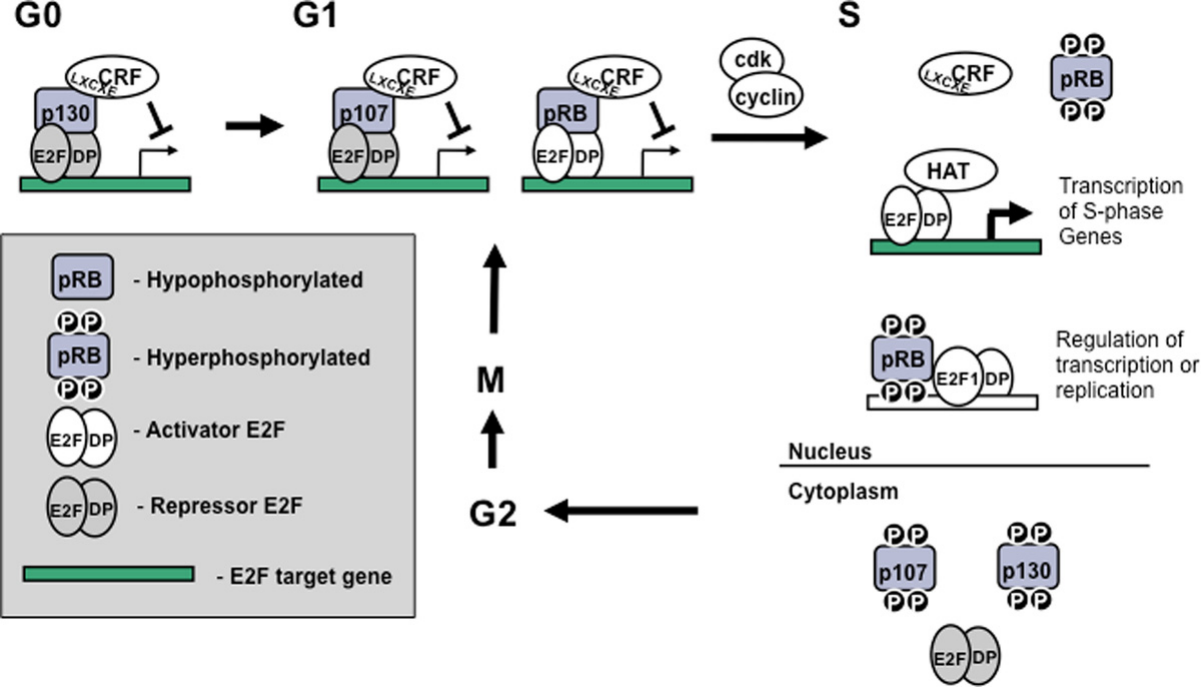
**Model of cell cycle entry control by pocket protein**. Beginning in the top left corner, quiescent cells repress transcription of E2F targets genes largely through the actions of p130. As cells progress into G1, complexes containing p107 and a repressor E2F such as E2F4 begin to replace p130. Furthermore, complexes of pRB and activator E2Fs such as E2F3 also become more abundant. Chromatin remodeling factors (CRF) are recruited to these complexes and mediate alterations to the chromatin environment, preventing transcription of E2F responsive genes. As a result, transcription of E2F target genes remains low until entry into S-phase. At the transition to S-phase, cyclin/CDK complexes phosphorylate the pocket proteins, dissociating them from the E2F/DP duplexes and transcription of E2F target genes proceeds through S phase. As part of this transition, the repressive heterochromatin changes that were present in G1 are reversed by the recruitment of new enzymes by the E2Fs, histone acetyltransferases (HAT) are examples of this type of enzyme. Another important change at the start of S-phase is the export of p130 and 107 proteins from the nucleus. At this point pocket proteins are thought to be relatively functionless until they are dephosphorylated and reactivated at the end of mitosis so that they can regulate transcription again during the next G1 phase.

In order to illustrate the roles of pocket proteins in cell cycle regulation we will begin by describing an idealized mammalian cell cycle (Figure 2). In this way, our goal is to summarize data from many experimental systems and condense it into a model that best captures our current understanding of RB family protein function throughout the mammalian cell cycle.

### Quiescence

**Figure 3:**
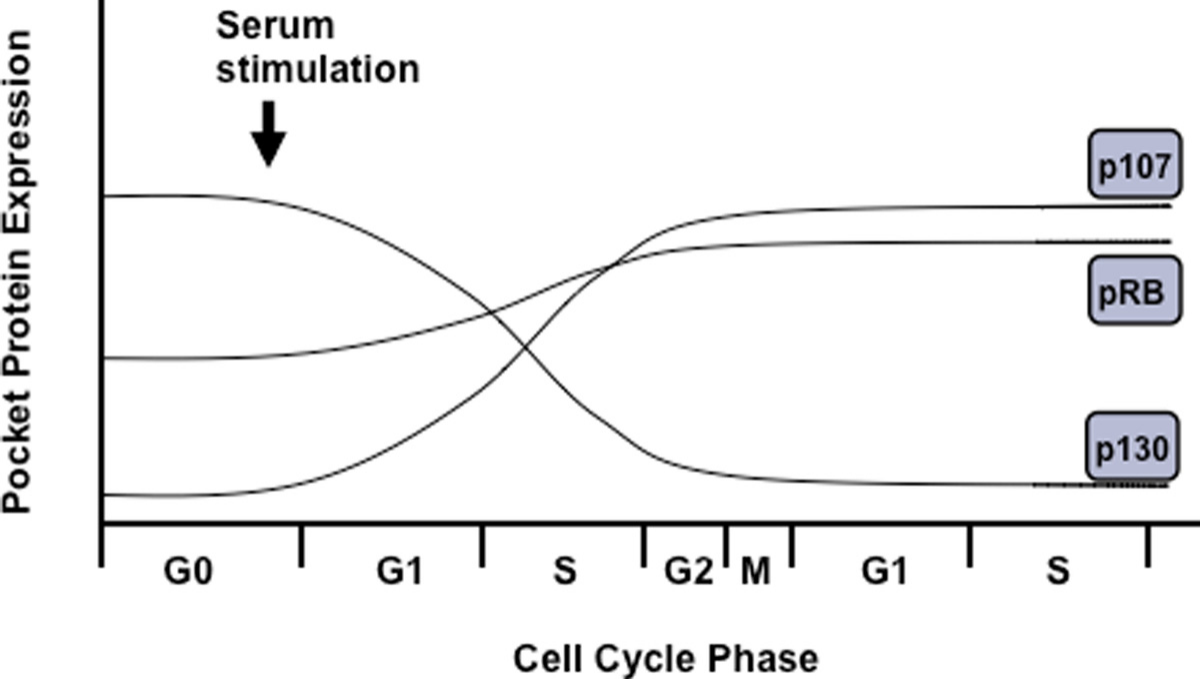
**Expression levels of pocket proteins throughout the cell cycle**. In G0, the most abundant pocket protein is p130. After cells are stimulated to enter the cell cycle expression of pRB and p107 are induced because they are E2F target genes themselves. At the same time these pocket proteins increase, the expression level of p130 begins to decline. In subsequent cell cycles pRB and p107 remain expressed at relatively constant levels, conversely, p130 is relatively inabundant under these growth conditions. These unique expression patterns offer clear, distinguishing characteristics of each pocket protein family member.

Often referred to as G0, quiescence is a resting state that is usually achieved through serum starvation of cells in culture. Of the three pocket proteins, p130 has the highest expression level in quiescent cells (Figure 3) and at this stage of the cell cycle the majority of E2F containing complexes contain p130 and E2F4 [42,43]. Recently it has been shown that p130 is part of a transcriptional repressor complex called DREAM, and it functions to repress E2F target genes during G0 [44]. Under these growth conditions pRB expression is low, but detectable in complex with E2Fs, while p107 is nearly undetectable [42,43]. In addition to E2F targets, pocket proteins also influence the expression of ribosomal and tRNA transcription in quiescence [45]. Both pRB and p130 are capable of repressing the transcription of rRNA genes by RNA polymerase I; a function that is not shared with p107 [46-48]. In addition, during G0 hypophosphorylated pRB binds to and represses TFIIIB, suppressing transcription by RNA polymerase III and reducing tRNA levels [49,50]. In contrast to rRNA transcription, regulation of tRNA expression has not been established for p107 or p130, it appears to be pRB specific. G0 has characteristically low levels of both rRNA and tRNA, which could be explained by pocket protein repression of RNA polymerase I and/or III [45]. The increase in rRNA and tRNA levels as the cell enters into the G1 phase has been proposed to involve phosphorylation of the pocket proteins to relieve this transcriptional repression [45]. While pRB has been found to be phosphorylated at G0 exit [51], a direct link between this phosphorylation and derepression of rRNA and tRNA genes remains to be shown.

### Progression of the cell cycle through G1 - regulation of E2Fs

It is not completely clear what differences in proliferative control separate G0 from G1, however, as discussed above, there are many differences in nucleic acid metabolism. The most distinguishing cell cycle marker of G0 compared to G1 is actually high level expression of p130 [52]. Nevertheless, cells are capable of halting proliferation in G1 in subsequent cell cycles when p130 levels are low, indicating that arrest can take place at this stage as well. In the context of an advancing cell cycle that has started in G0, G1 is best thought of as a transition to S-phase. The mechanisms that can induce an arrest at this point in response to signals like DNA damage or terminal differentiation require pocket protein function, but will be discussed later in the sections on cell cycle exit.

In G1, all three pocket proteins share the ability to interact with the E2F family of transcription factors. There are eight E2F family members and among these, E2Fs 1-5 are capable of binding to pocket proteins [53]. Each of these E2F proteins needs to heterodimerize with one of three DP family partners that are capable of binding to DNA [54]. The importance of the balance between E2Fs and pocket protein expression levels in cell cycle control is highlighted by experiments that demonstrate that over expression of E2Fs can overcome pocket protein dependent growth inhibition [55,56]. Conversely, knock out of E2F transcription factors can suppress ectopic proliferation in *Rb1 *deficient mouse embryos [57-59].

E2F family members 1-3 are referred to as the activator E2Fs because they induce transcription more potently from E2F responsive promoters than other E2F family proteins. E2Fs 4 and 5 are termed the repressor E2Fs because they have limited activation potential. The activator E2Fs associate exclusively with pRB [60] whereas p107 and p130 preferentially bind to the repressor E2Fs, E2F4 and E2F5 [42,61-63]. In addition, E2F4 is also detectable in complexes with pRB [42]. Because E2F4 and 5 lack a functional nuclear localization signal they rely on p107 and p130 to recruit them to the nucleus [64,65]. This further ensures a transcriptional repressor role for these complexes because neither p107, p130, E2F4 or E2F5 can efficiently localize to E2F responsive promoters alone.

The composition of E2Fs and RB-family proteins at cell cycle target genes in G1 is largely dictated by the expression patterns of the pocket proteins (Figures 2 and 3). In early G1, p130-E2F4 is most abundant on the promoters of E2F responsive genes, mediating transcriptional silencing of these genes [66,67]. In mid- to late G1, when p130 levels drop and p107 levels increase, p107 replaces it at E2F responsive promoters [67]. By late G1, pRB-E2F complexes, whose levels have been increasing throughout cell cycle entry also become more abundant [66]. At this point pRB is associated with activator E2Fs in a configuration that masks the E2F activation domain and prevents activation of transcription [32,68-70]. It is likely that pRB-E2F complexes are present at the promoters of E2F regulated genes, however, chromatin immunoprecipitations by different research groups have yielded varying results and this has left this as an open question [66,67]. Regardless of the exact mechanism by which each RB family protein works, it is clear that in G0 and G1, the pocket proteins cooperate to prevent the transcription of E2F regulated genes.

The E2F target genes that are subject to this regulation include cell cycle regulators such as cyclins A and E, the activator E2Fs, and pRB and p107 themselves [43,66,67,71]. Others include components of replication machinery such as the proliferating cell nuclear antigen and DNA polymerase α, as well as enzymes involved in nucleotide biosynthesis such as dihydrofolate reductase and thymidylate synthase [72,73]. How individual pocket protein/E2F complexes select target promoters beyond the E2F recognition sequence is not known. Some genes, such as b-Myb, are specifically regulated by p107 and p130 [43], while others, like p107 itself, are exclusively regulated by pRB [43,67,74]. Others still are reported to be occupied unselectively by all RB family members [66]. The concept of pocket proteins controlling cell cycle advancement through their interaction with E2Fs to negatively regulate transcription, at this stage of the cell cycle, is central to current thinking on RB family function. This is further underscored by the fact that viral oncogenes, such as E1A, are capable of inducing expression of E2F responsive genes by disrupting pocket protein/E2F interactions and this disrupting activity is, as discussed previously, required for viral oncogene induced cellular transformation [37].

A frequent companion to E2F regulation is the recruitment of transcriptional repressors (often that possess chromatin regulating enzymatic activity) to pocket protein-E2F complexes [53]. This allows specific enzymatic activities to be directed to very localized chromatin domains at these promoters. In the context of a cell cycle that begins in quiescence and continues uninterrupted to the initiation of DNA synthesis, it is not clear how important repressive modes of chromatin really are for cell cycle progression through G1. For example, cells from mice bearing a mutation in their pRB LXCXE binding site, that disrupts the interaction between chromatin regulators and pRB, have no defect in regulating progression through the G1 phase of the cell cycle [75]. However, deficiency for this interaction does compromise cell cycle exit [76]. As a result, regulation of chromatin by RB family proteins will be discussed later in the context of cell cycle exit.

### Progression of the cell cycle through G1 - regulation of cyclin dependent kinases

**Figure 4:**
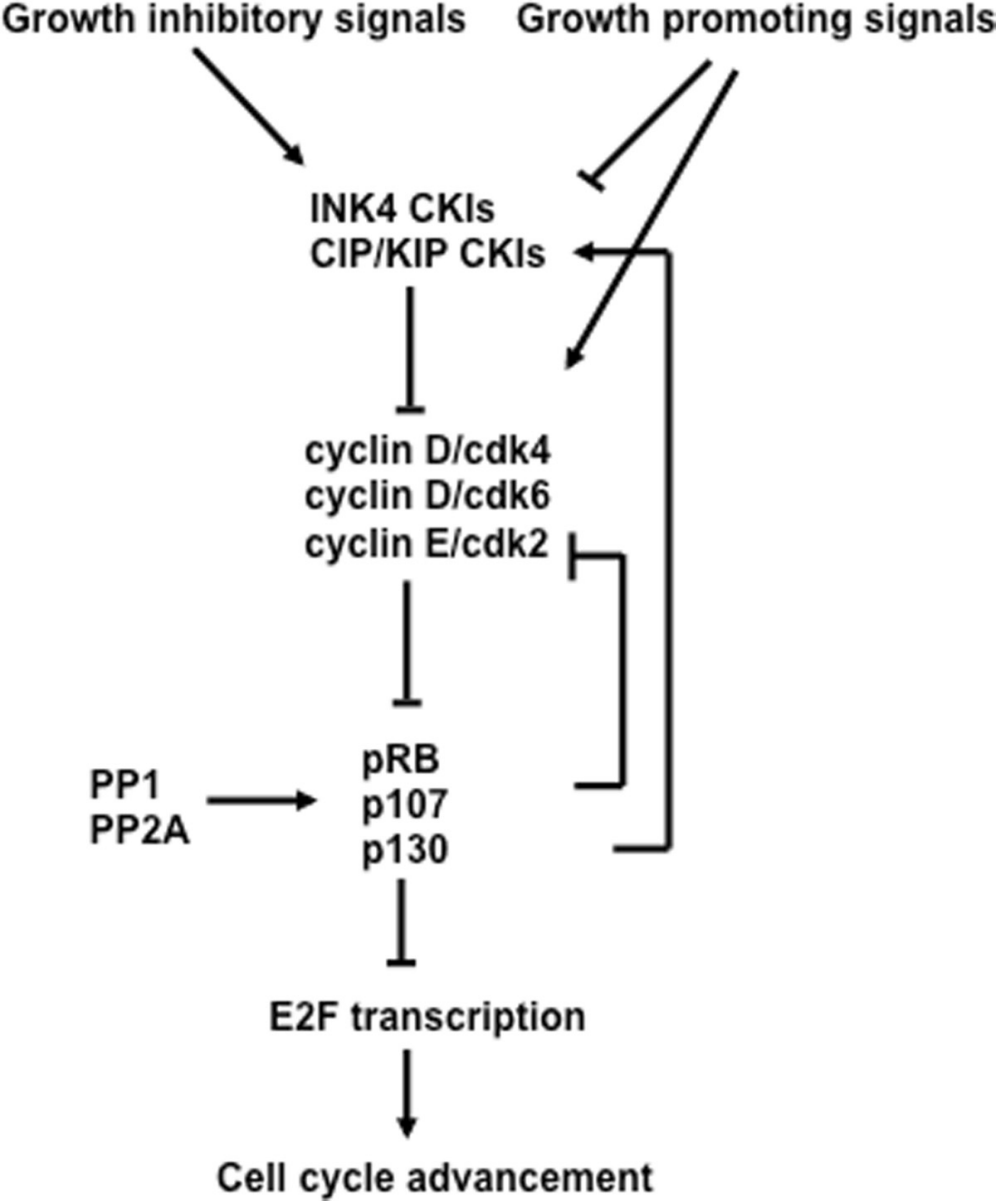
**The interrelationship of cell cycle regulatory molecules in G1**. Growth inhibitory and promoting signals impinge on the regulation of cyclin dependent kinase inhibitors (CKI). Growth promoting signals also directly lead to activation of cyclin dependent kinases (CDK) in G1. The cyclin dependent kinases serve to inactivate pocket proteins through phosphorylation leading to E2F transcriptional increases and cell cycle advancement. Cell cycle exit can be caused by activation of pocket proteins by phosphatases. In addition to blocking E2F transcription, recently activated pocket proteins also serve to negatively influence cyclin dependent kinase activity and positively influence CKI abundance. In this way, the regulatory molecules that control progression through G1 are extensively regulated by one another.

The ability of pocket proteins to interact with E2Fs is dependent on them being maintained in an under phosphorylated state [77]. The phosphorylation status of pocket proteins is often determined by their migration in SDS-PAGE, the slowest migrating forms are extensively modified and don't interact with E2Fs and this is described as 'hyperphosphorylated'. Alternatively, the fastest migrating forms are modified at very few positions and readily bind to E2F transcription factors, a state often referred to as 'hypophosphorylated'. It is imperative for cells to maintain cyclin dependent kinase activity at low levels until the end of G1. For this reason, discussion of the mechanisms that control cyclin dependent kinase activity are necessary for understanding pocket protein function during the G1 phase (Figure 4).

Cyclin dependent kinases are controlled at many levels from the assembly into complexes with cyclin subunits to regulatory phosphorylation that controls catalytic activity. However, the type of control that is most relevant to the activity of pocket proteins is at the level of cyclin dependent kinase inhibitor (CKI) proteins because they act immediately upstream of cyclin dependent kinases to block catalytic activity (Figure 4). There are two main classes of CKIs--the CIP/KIP family, which consists of p21^CIP1^, p27^KIP1 ^and p57^KIP2 ^and the INK4 family, which consists of p16^INK4A^, p15^INK4B^, p18^INK4C^, and p19^INK4D ^[78]. CIP/KIP family members contact both the cyclin and CDK subunits when they bind and inhibit kinase activity. They are not selective of cyclin dependent kinases and are able to inhibit any cyclin associated kinases that are found in G1. Alternatively, the INK4A members can bind only cdk4 and cdk6 with D-type cyclins [79].

As cells progress through the G1 phase, p27^KIP1 ^plays a key role in determining the onset of S-phase. In G1 p27^KIP1 ^expression levels are relatively high and the kinase activity of cyclin E/cdk2 is low and this prevents DNA synthesis from being triggered [79]. The first kinase complexes to become active in G1 are cyclin D/cdk4 or 6 and they begin to phosphorylate RB family proteins [77]. As cells approach the end of G1, partial phosphorylation of the pocket proteins allows some transcription by activator E2Fs that begins to stimulate production of cyclin E, and in turn creates more cyclin E/cdk2 kinase activity [77,79]. The most significant impediment to full activation of this kinase complex is the interaction with and inhibition by p27^KIP1 ^[79]. This is ultimately overcome by cyclin E/cdk2 phosphorylating p27^KIP1 ^resulting in it being targeted for degradation and allowing for full activation of the kinase and ultimately entry into S-phase. Because of p27^KIP1^'s pivotal role in regulating kinase activity to keep pocket proteins active in G1, it is subjected to extensive regulation itself. As an example of p27^KIP1^'s regulation, the growth promoting proto-oncogene c-myc has the ability to regulate cyclin/CDK activity through p27^KIP1^. Cells lacking c-myc have a decreased growth rate due to increased p27^KIP1 ^and an associated decrease in cyclin/CDK activity [80], whereas expression of c-myc results in repression of p27^KIP1 ^transcription [81].

In addition to the CIP/KIP and INK4 inhibitors, the RB family members p107 and p130 also behave as cyclin dependent kinase inhibitors by directly binding to cyclin/CDK complexes [40,82-85]. The interaction between p107 and p130 and cyclin/cdks allows them to inhibit kinases to suppress growth [21,39,41], a function that is not shared by pRB [86]. In fact, p107 is as potent an inhibitor as p21^CIP1 ^[86]. For example, p107 and p130 can use their cyclin dependent kinase inhibitory activity to regulate the progression through G1 as induction of p130 inhibits cyclin E/cdk2 and induces p27^KIP1 ^levels [87]. Two independent regions have been shown to mediate p107 and p130's ability to inhibit cyclin A/cdk2 and cyclin E/cdk2 (Figure 1B). The first is the spacer region between the A and B pockets, and the second is a highly conserved region in the N-terminus of p107 and p130 [39,41]. It is the combination of these dual domains that allows p107 and p130 to inhibit kinase activity. While the N-terminal region in p107 can inhibit cyclin/CDK complexes, it has a weak affinity for cyclin/CDK binding [86]. In contrast, the binding site in the spacer region readily interacts with these kinases, but cannot inhibit cyclin/CDK activity [86]. Mutagenesis experiments using p130 showed that deletion of the spacer region prevents binding of cyclin E and cyclin A but that this mutant can still suppress cell growth, indicating that binding and inhibition of cyclin/CDKs are mediated by different regions of the protein [39]. While both p107 and p130 can act as cyclin dependent kinase inhibitors when overexpressed, the circumstances where this aspect of p107 and p130 function is most critical *in vivo *has remained elusive.

It is known that pRB does not possess a kinase inhibitory domain that is analogous to the other family members (Figure 1B); however, it has the means to control cyclin/CDK activity through inhibitor proteins. Specifically, it can interfere with the targeted degradation of p27^KIP1 ^to maintain G1 kinase activity at low levels at a number of places in the degradation pathway [88,89]. Maintenance of p27 expression can be accomplished when pRB acts as a scaffold that interacts with APC^Cdh1 ^and Skp2, simultaneously targeting Skp2 for ubiquitin-mediated degradation [89]. Because Skp2 is an adaptor that is necessary for targeting p27^KIP1 ^for degradation, this increases p27^KIP1 ^levels and results in a net decrease of G1 CDK activity. Furthermore, pRB's ability to interact with Skp2 also allows it to compete for binding with phosphorylated p27^KIP1 ^[88]. Therefore, pRB also directly blocks ubiquitination of p27^KIP1 ^to maintain inhibition of CDK activity. Studies have also shown that p107 can decrease Skp2 levels and increase p27^KIP1^, delaying S-phase entry [90,91]. This suggests that p107 may also possess the ability to regulate p27^KIP1 ^degradation. It should be emphasized that comparatively little is known about how pocket proteins regulate p27^KIP1 ^expression in comparison with E2F regulation. It remains unclear as to how important this control mechanism is, even though some experiments have suggested that it may be as critical for controlling progression through G1 as E2F regulation [88,89].

### The G1 to S-phase transition

Entry into S-phase is an important commitment for mammalian cells. Once DNA replication has begun the cell cycle must progress until cell division is complete. The consequence of the cell cycle slipping backwards into G1 after initiating replication is genome instability and possibly cancer. As such, the commitment step to initiate DNA replication possesses many features that ensure cells progress exclusively from G1 to S-phase and not in the reverse direction. Pocket proteins play a key role in this mechanism.

Critical events that ensure G1 to S progression is unidirectional include feed forward loops that increase cyclin dependent kinase activity, pocket protein phosphorylation, as well as the proteolytic degradation of cyclin dependent kinase inhibitors such as p27^KIP1 ^[79]. As intimated in the previous section on progression through G1, cyclin dependent kinase activity rises towards the end of G1 as does E2F transcriptional activity [92]. Since cyclin E/cdk2 kinases are the most active kinase complex at the G1 to S-phase transition, their kinase activity is thought to be most important in triggering this transition. Furthermore, cyclin E is an E2F target gene and the best available evidence has suggested that among pocket proteins, pRB is primarily responsible for regulating its expression [43,66]. This is in part because cyclin E is deregulated in *RB1 *deficient cells whereas loss of the other pocket proteins don't affect its expression [43,93], but also because only pRB negatively regulates the activator E2Fs that are essential to induce transcription of the cyclin E gene [92]. For these reasons the feed forward loop that ensures that cells advance to S-phase irreversibly relies on cyclin E/cdk2 phosphorylation of pRB to release activator E2Fs that transcribe more cyclin E and generate more kinase activity towards pRB, leading to more free E2Fs and ultimately even more cyclin E (Figures 2 and 4). At the same time p27^KIP1 ^is also phosphorylated and targeted for degradation, allowing cyclin E/cdk2 to become maximally active [79]. Once cells have committed to S-phase progression and initiated DNA synthesis, cyclin E/cdk2 will phosphorylate other cyclin E subunits and this will target them for degradation, thus bringing the surge in cyclin E/cdk2 activity to an end. The other pocket proteins are also phosphorylated in a manner that is analogous to pRB releasing their associated E2F transcription factors [21]. Furthermore, a portion of these pocket proteins are exported from the nucleus where they go on to form abundant complexes with repressor E2Fs in the cytoplasm [94,95]. This is particularly true of p107 whose expression is greatly elevated by cell cycle entry because it is an E2F target gene. Conversely, p130 expression continues to decline as the cell cycle advances in part due to its proteolytic degradation [96], consequently, cytoplasmic complexes between it and E2Fs are relatively inabundant [21].

The point of no return for cell cycle entry has often been termed the 'restriction point'. Recent work measuring E2F transcription in single cells offers fresh insight into the importance of the pRB-E2F regulatory interaction in G1-S control [97]. This report suggests that release of E2F from pRB control by phosphorylation coincides specifically with the point in which cells are obligated to complete the rest of the cell cycle. Therefore to thoroughly understand this intricate step in the cell cycle, it is important to review the specifics of how exactly phosphorylation controls pRB-E2F interactions.

The RB protein contains 16 consensus cyclin dependent kinase phosphorylation sites that span the spacer region as well as both N- and C-terminal regions, but appear to be largely excluded from the small pocket [98-100]. Early in the G1 phase of the cell cycle, D-type cyclins along with cdk4 and cdk6 phosphorylate pRB, prior to cdk2 activation [77,79]. Late in G1, cyclin E/cdk2 further phosphorylates pRB, completely disrupting its ability to bind E2F complexes. The sequential phosphorylation of pRB by ckd4/6 followed by cdk2 is necessary because cdk4/6 alone is unable to completely phosphorylate pRB whereas cdk2 cannot use unphosphorylated pRB as a substrate [101]. Mutation of individual phosphorylation sites in pRB does not disrupt the ability to block cell proliferation, indicating that no single site regulates pRB and E2F binding [99,100]. Instead it has been shown that the majority of phosphorylation sites on pRB need to be modified to abrogate E2F binding. Furthermore, it has been demonstrated that phosphorylation of multiple regions such as the spacer and C-terminus together are necessary for displacement of E2Fs from pRB [99]. Analysis of CDK phosphorylation of p107 and p130 is much less extensive than for pRB. However, databases of phosphoproteomic data such as PhosphositePlus suggest that p107 and p130 are also phosphorylated in similar regions surrounding the pocket domain [102].

Once free of regulation from pRB, activator E2Fs associate with histone acetyl transferase enzymes such as p300 [103] (Figure 2). These enzymes mediate acetylation of histone H3 and H4, allowing for transcription of E2F target genes to ensure sufficient supplies of nucleotides and other factors necessary for completing S phase of the cell cycle [103].

### Progression through S-phase

Our understanding of pocket proteins in cell cycle control is strongly influenced by the idea that pRB and its family members are inactivated at the start of S-phase and therefore functionless until dephosphorylated and reactivated at the end of mitosis. While this paradigm is supported by a number of lines of evidence, exceptions to this rule are beginning to surface.

A number of reports indicate that pRB and E2F1 complexes are either resistant to cyclin/CDK phosphorylation, or that they exist in S-phase, thus implying resistance to control by CDKs [104-106]. This apparent paradox in E2F regulation can be explained by the fact that pRB possesses two mechanisms to interact with E2F transcription factors and one of them is unique to E2F1 and is resistant to phosphorylation [107,108] (Figure 1B). While the function of this complex is not entirely clear, pRB bound to E2F1 in its CDK resistant configuration has altered DNA binding specificity compared with canonical pocket protein-E2F complexes [107]. One possibility is that hyperphosphorylated pRB bound to E2F1 represses transcription of select, pro-apoptotic E2F target genes such that E2F1's apoptotic function can be inhibited while its ability to drive proliferation is activated [108].

When cells experience DNA damage during S-phase they need to arrest replication and repair the damage before proceeding. This S-phase checkpoint function has been shown to be absent in *RB1 *deficient cells, defining a role for pRB in this process [109]. Following DNA damage in S-phase, pRB is dephosphorylated and this allows it to mediate the repression of cyclin A transcription. This causes a subsequent decrease in cdk2 activity and decreased proliferating cell nuclear antigen tethering to chromatin, thereby disrupting DNA replication [110,111]. Furthermore, it has also been shown that pRB can physically localize to replication origins in S-phase to arrest DNA synthesis, although the molecular mechanism by which it blocks synthesis is unknown [112]. While it is possible that phosphorylated pRB in association with E2F1 may mediate some of these S-phase effects, protection of pRB from phosphorylation has also been described. For pRB to localize on chromatin at replication origins during S-phase, it has been suggested that a small proportion of pRB is protected from cyclin dependent kinases at these regions. This is supported by the fact that inhibition of the phosphatase PP2A causes pRB to be lost from these regions of the genome [112]. Furthermore, it has been shown that pRB is acetylated in response to DNA damage and this modification blocks recognition of pRB by cyclin dependent kinases [113]. Thus, in addition to being inducibly dephosphorylated during S-phase [114], pRB can also be marked to remain active in the face of high levels of cyclin dependent kinase activity [113,115]. Taken together, this narrative on pocket protein function in S-phase reveals a role for these proteins in arresting cell cycle advancement during this phase. In addition, some details of the mechanism that activates pRB and allows it to block DNA synthesis or inhibit apoptosis are emerging.

### The role of pocket proteins in navigating mitosis

In addition to their roles in the G1 and S phases, the pocket proteins have also been implicated in controlling events during mitosis. In previous sections it has been discussed that E2F target genes are induced to function in S-phase. However, some E2F target genes are induced later and function in G2 or mitosis. Thus, misregulation of E2F target genes early in the cell cycle because of defects in pocket protein function can be manifested later in mitotic errors. For example, pRB deficient cells have a characteristic over expression of mitotic checkpoint genes Emi1 and Mad2, which are both E2F responsive targets. This over expression delays the progression through mitosis and results in binucleated, aneuploid, and polyploid cells [116,117]. Furthermore, in nocodazole arrested cells, defective licensing of DNA replication in *RB1 *deficient cells leaves them more prone to re-replicate DNA following failure to progress through mitosis [118,119]. These cells ultimately resume proliferating with increased ploidy.

Beyond these E2F dependent effects on mitosis, non-E2F dependent roles in controlling chromosome architecture are also performed by RB family proteins. Deficiency for all three pocket proteins results in mitotic errors caused by faulty chromatin structure in pericentromeric regions [120]. These mitotic errors most frequently cause cells to become tetraploid. It has also been suggested that this role in chromosome packaging during mitosis is mostly carried out by pRB since a knock in mouse strain that is defective for LXCXE interactions also has this defect even though p107 and p130 proteins are functional. On a molecular level defective pRB function in mitosis prevents condensin II loading onto mitotic chromosomes [121-123]. This results in defective chromosome congression at the metaphase plate and merotelic attachments between microtubules and centromeres. Ultimately these defects manifest in lagging anaphase chromosomes [122,123]. Similar mitotic phenotypes have been observed in cells deficient for all pocket proteins suggesting that this function is primarily carried out by pRB [124].

Lastly, at the end of mitosis pocket proteins are dephosphorylated to regulate E2Fs and guide progression in the ensuing G1 phase. Dephosphorylation has been extensively studied for pRB and while it has been thought to be primarily mediated by protein phosphatase 1 (PP1), reports of protein phosphatase 2 acting on pRB have emerged more recently [125-127]. Intriguingly, a direct contact site for PP1 on pRB has been identified and it corresponds to the same short peptide sequence used by cyclin/CDK complexes to bind and phosphorylate pRB [128] (Figure 1B). What this means is that the principal phosphatases and kinases that modify pRB must compete with one another for substrate access. In functional terms this creates an additional level of regulation in which PP1's ability to dephosphorylate is accelerated because it simultaneously inhibits CDK access to pRB as a substrate [128]. This competition mechanism is likely unique to pRB because the cyclin/CDK binding sites in p107 and p130 lack an embedded RVXF motif used by PP1 [128]. Importantly, PP1-pRB complexes are most abundant in mitosis, suggesting that this mechanism is part of pRB activation at mitotic exit [129]. In contrast, only protein phosphatase 2 has been shown to dephosphorylate p107 and p130 [130,131].

## The role of pocket proteins in cell cycle exit

The preceding sections of this review have highlighted the roles played by RB family proteins in a typical mammalian cell cycle. In addition to ensuring fidelity in replication and cell division, they are also critical to orchestrating a cell's exit from proliferation. This occurs either as a checkpoint to repair DNA damage before resuming proliferation, or it can be more permanent as in terminal differentiation during development where cells ultimately move into a G0 like state. The ensuing sections are meant to provide a basic overview of how cell cycle exit is controlled by pocket proteins. A number of examples of cell cycle exit are used to highlight how the different pocket proteins participate in this process. Outlining the intricacies of all known RB-dependent cell cycle arrest events is beyond the scope of this review. The following examples were selected because they offer some of the best insight into the unique roles of the different RB-family proteins during this process.

### Activation of pocket proteins during a reversible cell cycle arrest

As explained in the earlier section on progression through G1, this is the cell cycle phase where pocket proteins are best able to influence cell cycle decisions. For this reason RB-family dependent cell cycle arrest in G1 requires the inhibition of cyclin dependent kinases to keep pocket proteins underphosphorylated and active. The members of the INK4 and CIP/KIP families of cyclin dependent kinase inhibitors play a crucial role in this activation step (Figure 4). One mechanism of pocket protein activation is in response to DNA damage. Following DNA double strand breaks, p53 is activated and induces expression of p21^CIP1 ^[14]. This inhibitor then blocks activity of cyclin dependent kinases and prevents the phosphorylation of RB-family proteins. This will ensure that cells remain in the G1 phase of the cell cycle because the pocket proteins will remain hypophosphorylated. If DNA damage occurs in S-phase, as described in a previous section of this article, phosphatases are also necessary to dephosphorylate RB-family proteins to ensure they are capable of binding E2F transcription factors [114]. DNA damage offers one of the best examples of how an exogenous stimulus can communicate growth arrest signals to central regulators of proliferation like the pocket proteins.

Another informative example of an extra cellular signal leading to inactivation of cyclin dependent kinases and activation of RB family proteins is during an arrest that is stimulated by the growth suppressing cytokine TGF-β. In particular, this example demonstrates differences among pocket proteins and how they contribute to a cell cycle arrest. Like DNA damage, TGF-β signaling directly induces expression of cyclin dependent kinase inhibitor proteins [132]. In epithelial cells the INK4 protein p15^INK4B ^is induced by TGF-β [133]. Since INK4 proteins can only bind and inhibit activity of cyclin D associated kinases, part of the inhibitory mechanism involves the displacement of CIP/KIP proteins such as p27^KIP1 ^from D-type cyclins so that they are free to inhibit cyclin E or cyclin A associated kinases [132]. TGF-β signaling also inhibits degradation of p27^KIP1^, further ensuring that this inhibitor molecule increases in abundance [134]. TGF-β blocks cell cycle advancement in the G1 phase [135], for this reason its actions serve to arrest cells that have already progressed to G1 and then maintain pocket proteins in an active, hypophosphorylated state. In this circumstance, pRB is required to repress expression of E2F target genes [76]. Furthermore, a complex containing Smad2/3, p107, and E2F4/5 is recruited to the c-Myc promoter to repress its transcription in response to TGF-β [136]. Intriguingly, this co-Smad role for p107 and E2F4/5 can be activated by TGF-β signaling at any stage of the cell cycle. This suggests that individual pocket proteins have highly specialized roles in responding to cell cycle arrest signals. Furthermore, recent evidence also suggests non-E2F dependent roles for pRB in TGF-β cell cycle arrest, further emphasizing the diverse nature of pocket protein function in this cell cycle arrest paradigm [137].

TGF-β signaling induces proliferative arrest in many cell types. The cyclin dependent kinase inhibitors that it regulates vary between these cells. Each of p15^INK4B^, p21^CIP1^, and p57^KIP2 ^are transcriptionally upregulated individually, or in combination, depending on the cell type in question [132]. This points to the importance of cyclin dependent kinase inhibition in maintaining pocket proteins in an active state in G1 (Figure 4). Further evidence of the importance of the whole RB-family in cell cycle arrest is exemplified by the ectopic expression of the p16^INK4A ^protein in cells deficient for different combinations of pocket proteins. Loss of pRB, or the combined loss of p130 and p107, both abrogate p16^INK4A ^induced cell cycle arrest, revealing a broad requirement for pocket proteins in responding to cyclin dependent kinase inhibition [138]. These experiments suggest that collectively the pocket protein family participates in cell cycle arrest that is stimulated by exogenous signals such as TGF-β.

### Recruitment of chromatin regulating factors by pocket proteins in senescence

The TGF-β arrest mechanism described above is reversible, implying that once the growth arrest signal is removed cell proliferation can resume. However, prolonged stimulation by TGF-β, or chronic genotoxic stimuli such as DNA breaks or telomere attrition, can cause cells to enter a permanent arrest known as senescence [139]. This subsection of the review will discuss the functions of RB-family proteins in establishing a permanent cell cycle arrest. RB-family dependence in senescence is well supported by experiments using mouse embryonic fibroblasts deficient for all pocket proteins, because these cells fail to senesce in response to oncogenic Ras or ectopic expression of cyclin dependent kinase inhibitors [140-142]. Similarly, disruption of just pRB and p107 abrogates Ras-induced senescence and results in uncontrolled proliferation. Furthermore, loss of pRB in already senescent cells leads to a reversal of this cell cycle arrest and a resumption of proliferation[142,143]. Thus, these studies collectively emphasize roles for the whole RB-family, as the phenotypes of individual gene knock outs can't account for deregulated proliferation found in triple deficient cells.

As above, cell cycle arrest in senescence is initiated through increased expression of cyclin dependent kinase inhibitors; in particular p16^INK4A ^expression is induced in senescence. Once activated, the pocket proteins can repress transcription of E2F targets and cause the cell cycle to arrest. Since this also happens in reversible cell cycle arrest, there has been considerable interest in mechanisms that establish long term gene silencing at E2F targets [144]. As described earlier, the pocket proteins contain a well-conserved binding cleft that mediates interactions with LXCXE containing proteins involved in transcriptional repression [53]. Importantly, RB-family proteins can interact with E2Fs and LXCXE proteins simultaneously. This allows pocket proteins to recruit LXCXE containing proteins, particularly ones that have chromatin regulating activity, to the promoters of E2F-responsive genes where they are responsible for condensation of chromatin and inhibition of transcription. Chromatin remodeling enzymes that have been identified to bind to RB-family proteins include SWI/SNF remodeling factors such as BRG and BRM, histone deacetylases (HDAC1, 2 and 3), and histone methyltransferases (Suv39h1 and 2), among others [145-152]. The diversity of these interacting proteins allows the pocket proteins to exert widespread effects on chromatin structure. For example, pRB has been associated with decreased histone acetylation and increased H3K9 and H3K27 trimethylation at the promoters of E2F target genes [153]. By mediating histone deacetylation, as well as methylation, pRB contributes to the formation of heterochromatin [153]. Taken together, this suggests that pRB may facilitate a reversible arrest by deacetylating histones and directing a more permanent arrest through histone methyltransferases and gene silencing. Indeed, key cell cycle E2F target genes are reported to be silenced by pRB and a side by side comparison revealed that p107 and p130 are dispensable for regulation of these same genes [154]. Furthermore, cells from a gene targeted mutant mouse strain in which pRB is unable to interact with LXCXE motif containing proteins demonstrates that pRB can support the initial entry into senescence, but can't permanently silence transcription. Histones at E2F target genes fail to be methylated, and transcription of these targets is activated by ectopic E2F expression [155]. Assembly of heterochromatin is often undertaken in promyelocytic leukemia (PML) bodies, and pRB and E2Fs are associated with PML in senescence [156]. This places pRB in the heart of the mechanism that assembles heterochromatin during senescence.

The ability to recruit chromatin remodeling enzymes to the promoters of E2F target genes is not limited to pRB alone, as both p107 and p130 can repress E2F activity through recruitment of histone deacetylases [157,158]. However, there is less evidence for p107 or p130 recruiting histone methyltransferases, consistent with pRB having a unique role in gene silencing. Work by Shamma et al. offers an example of p130 recruiting Suv39h1 to E2F gene promoters in senescence to methylate histones in the absence of pRB [159]. Other reports have demonstrated that the mammalian DREAM complex containing p130 plays a key role in the maintenance of senescence [160,161]. In this scenario Dyrk kinases phosphorylate the Lin52 subunit in the DREAM complex and this facilitates assembly with p130 and gene silencing of E2F targets in the maintenance of senescence.

Beyond the regulation of transcription at specific E2F target genes, senescent human cells are often characterized by senescence associated heterochromatic foci (SAHF) [162]. These are heterochromatin bodies containing individual chromosomes that have been shown to be associated with pRB mediated repression of E2F target genes and their formation is dependent on pRB function [163,164]. These heterochromatin structures have many features in common with inactive X-chromosomes suggesting that pRB function in senescence is key to triggering large scale chromatin changes as a downstream consequence [165].

## Conclusions

The sections described above are in no way meant to recap all of the important contributions in pocket protein research but rather to provide an overview of the advances that have shaped our understanding of pocket protein function in cell cycle regulation. There are many fundamental questions in proliferative control that remain to be answered. From this work, some basic principles of pocket protein function may be emerging. For example, the sections on G1 to S-phase transition and cell cycle exit both illustrate the role for pRB in transition from one phase to another, or from proliferation to arrest. In this sense pRB is less critical in a population of rapidly proliferating or quiescent cells, but becomes more important when cells are faced with decisions to enter or exit a proliferative state. In general, the roles of p107 and p130 proteins are less central to cell cycle decisions. Instead, the abundance of p130 and the regulation of its assembly into the DREAM complex suggests that its role is most crucial in the maintenance of prolonged cell cycle arrest such as in quiescence or senescence. This may offer some insight into why the *RB1 *gene is mutated in cancer, but the *RBL2 *gene (encoding p130) is generally spared. While pRB's and p130's roles and periods of activity are becoming clearer in relation to one another, p107 remains an enigma. Comparisons between cells deleted for the genes encoding all pocket proteins and those deleted for pRB and p130 together demonstrate a clear contribution of p107 to the overall role of RB-family proteins in cell cycle control. However, there are few circumstances in which p107 has a biochemical function that isn't replaceable by other pocket proteins. Responsiveness to TGF-β and suppression of c-Myc transcription appears to be its sole unique function [136], and even this has yet to be demonstrated to be essential for TGF-β growth suppression. Future work on RB-family proteins will need to continue to uncover unique roles for these proteins, only then will we truly understand how they collectively regulate cell proliferation.

## Abbreviations

CDK: Cyclin dependent kinase; CKI: Cyclin dependent kinase inhibitor; DREAM: DP: RB-like: E2F: And MuvB; E2F: E2 promoter binding factor; HDAC: Histone deacetylase; LXCXE: Leucine-any amino acid-cysteine-any amino acid-glutamate peptide motif; PML: Promyelocytic leukemia; PP1: Protein phosphatase one; RB: Retinoblastoma; SAHF: Senescence associated heterochromatic foci; TGF-β: Transforming growth factor beta

## Competing interests

We study RB-family proteins in cell cycle control and some of our publications are cited in this review. We have endeavored to be as objective as possible in reviewing the literature, but we encourage readers to draw their own conclusions.

## Authors' contributions

Both SAH and FAD contributed to researching and writing this article. All authors read and approved the final manuscript.
